# Validation of an Instrumental Device to Estimate the Risk of Falls and Frailty in Older People

**DOI:** 10.3390/s26082472

**Published:** 2026-04-17

**Authors:** Eva Martí-Marco, Enrique J. Vera-Remartínez, Aurora Esteve-Clavero, Irene Carmona-Fortuño, Martín Flores-Saldaña, Jorge Vila-Pascual, Malena Barba-Muñoz, María Pilar Molés-Julio

**Affiliations:** 1 Department of Nursing, Faculty of Health Sciences, Universitat Jaume I, 12006 Castellón, Spain; al389241@uji.es (E.M.-M.);; 2 Universitary Hospital of La Plana, 12540 Villarreal, Spain; 3 General Hospital of Castellón, 12004 Castellón, Spain; 4 Artana Health Centre, 12527 Castellón, Spain

**Keywords:** accidental falls, fragility, older adults, validation study

## Abstract

**Highlights:**

**What are the main findings?**

- The Oldfry instrumental device demonstrates high reliability, sensitivity, and agreement for detecting fall risk and frailty in institutionalized older adults.
- Measurements obtained with Oldfry show a strong correlation with the reference clinical tests Timed Up and Go (TUG) and Short Physical Performance Battery (SPPB).
- The device improves the detection of frailty and mild fall risk compared with traditional manual assessment.

**What are the implications of the main findings?**

- The use of instrumental devices such as Oldfry enables objective, rapid, and accurate assessment of fall risk and frailty in residential care settings.
- Early identification of deficits in balance, mobility, gait, and muscle strength facilitates the planning of personalized physical activity interventions.
- The implementation of structured exercise programs supported by this type of technology may contribute to improving functional autonomy and quality of life in older adults.

**Abstract:**

**Objective**: To validate the Oldfry instrumental device for efficiently detecting the risk of falls and frailty in older adults. **Design and Methods**: An observational, analytical, cross-sectional, multicenter, non-randomized study to validate an instrumental device. It was conducted in several nursing homes for the elderly in the province of Castellón, Comunidad Valenciana, Spain, from February to April 2024. The estimated necessary sample size was 149 people. Specific selection criteria and voluntary acceptance to participate in the study were established. Sociodemographic, anthropometric, and other variables such as fall history in the past year were collected. A descriptive and comparative analysis of the variables was performed. The validity and reliability of the device in its measurements were determined to compare the results of the Timed Up and Go (TUG) test and the Short Physical Performance Battery test (SPPB), with respect to the Oldfry instrumental device. Informed consent was obtained from all participants, and the study was approved by the Bioethics Committee of the University Jaume I. **Results**: The sample consisted of 151 participants with a median age of 84 years (IQR [78.0–91.0]), comprising 39.10% men and 60.90% women, 65 years of age or older. Oldfry presents a sensitivity of 45.90% and a specificity of 72.7% for the risk of falls with a correlation R: 0.773 and an ICC concordance: 0.821. For frailty assessment, it shows a sensitivity of 91.90% and a specificity of 9.10% with an R: 0.854 and ICC: 0.805. **Conclusions**: This device has proven to be an effective tool for detecting both the risk of falls and frailty in older adults residing in institutions, showing high levels of reliability, sensitivity, and high concordance and correlation in both measurements. Future studies are anticipated to evaluate the benefits of this application.

## 1. Introduction

Falls pose a significant challenge to public health, as they represent the second leading cause of unintentional injury-related deaths globally. According to data from the World Health Organization (WHO) published in 2021, 684,000 people lose their lives annually due to falls, with individuals over the age of 60 being the most affected. Furthermore, medical attention following falls becomes crucial as 37.3 million people receive treatment each year [1]. In Spain, of the 8.1 million elderly individuals, 4.3 million falls are recorded annually, with 60% occurring at home [2].

Frailty is a clinical-biological syndrome with multiple manifestations but no specific symptoms. The term originated in the 1980s with [3]. There are multiple methods for screening frailty, which leads to considerable variability in prevalence estimates, ranging from 4% to 59.1% worldwide and from 8.4% to 20.4% in Spain; in contrast, the prevalence of pre-frailty is more consistent, ranging from 41.8% to 48.5% [4].

Frailty is a risk factor for falls in older adults, making it crucial for health professionals to understand the relationship between frailty and falls. Therefore, it is necessary to find tools to measure frailty and fall risk to prevent these accidents, manage frailty, and improve the quality of life for the elderly [5]. It is worth noting that institutionalized individuals are at higher risk of falls [6]. The high incidence of falls increases socioeconomic costs globally, often being a preventable factor. Hence, it is vital to focus on interventions that reduce the frequency of these events [7].

Validated tests, such as The Timed Up & Go (TUG), can quickly assess fall risk in older adults. Its simplicity and speed allow various health professionals to use it, speeding up care and improving the quality of life by identifying deficiencies [8]. Other validated tests, like the Guralnik Test known by its acronym SPPB (Short Physical Performance Battery), evaluate frailty in older adults [9].

Smart applications are widely used to collect data on balance parameters, gait speed, and muscle strength, enabling the monitoring of fall risk and/or frailty in older adults [10]. These parameters can help adopt timely interventions to improve their quality of life [11].

In this context of growing awareness about the importance of preventing falls in older adults, implementing instrumental devices could enhance the quality of life for the elderly through early detection and timely intervention in frailty and fall risk [12].

The successful integration of these applications into clinical practice could yield substantial benefits, allowing for fall prevention and frailty assessment, and adapting planned interventions [13,14,15].

Comparative studies conducted in the field of geriatrics indicate that the use of inertial sensors, such as accelerometers and gyroscopes, provides objective measurements of patients’ movement and balance, offering quantifiable information about their risk of falls. These technological tools have demonstrated that they can match or even surpass the predictive capacity of traditional clinical scales and expert assessments, which rely heavily on the evaluator’s subjective judgment. Unlike manual assessments, which exhibit inter-observer variability and limitations in reproducibility, instrumented devices provide accurate, consistent, and repeatable data, allowing for more rigorous and continuous monitoring of factors associated with fall risk. Therefore, the integration of these technologies not only increases measurement objectivity but also contributes to improving the early identification of at-risk individuals, thereby optimizing the planning of preventive interventions and reducing the incidence of falls in vulnerable populations [16,17].

In contrast to existing inertial sensor-based systems, which often focus on isolated parameters or single functional tasks, the Oldfry device (BLAUTIC, Valencia, Spain) has been designed to provide a more comprehensive and clinically applicable assessment. Many current approaches are limited by their focus on specific metrics such as gait speed or balance alone, or by their lack of direct correspondence with standardized clinical tools, which reduces their usability in routine practice.

Furthermore, some systems require complex setups or are not easily adaptable to real-world clinical and residential environments, limiting their scalability and practical implementation.

In this context, Oldfry aims to overcome these limitations by offering an integrated and streamlined evaluation that combines the assessment of fall risk and frailty—two closely related dimensions in older adults—within a single standardized protocol. Its ability to generate objective, consistent, and reproducible measurements reduces inter-observer variability and enhances clinical decision-making. By directly aligning sensor-derived data with established clinical scales such as TUG and SPPB, the system facilitates interpretation and supports its incorporation into routine geriatric assessment.

Unlike our previous study published in Sensors (2025) [18], which focused on identifying clinical and functional factors associated with fall risk and frailty using the Oldfry device, the present study is specifically designed as a validation study. Although both studies are based on the same cohort, the analytical approach is fundamentally different. The previous work explored associations between clinical and functional variables, whereas the current study evaluates the diagnostic performance, reliability, and agreement of the device in comparison with established clinical reference tests such as TUG and SPPB. Therefore, this work provides methodological evidence regarding the accuracy and reproducibility of the device, representing a complementary and necessary step toward its clinical implementation.

Therefore, our objective is to validate the instrumental device Oldfry, designed to efficiently detect fall risk and frailty in older adults.

## 2. Materials and Methods

### 2.1. Study Design, Setting, and Scope

A multicenter validation study with an observational, analytical, cross-sectional, and non-randomized design was conducted to determine the usefulness of an instrumental device for identifying fall risk and frailty in institutionalized older adults. This investigation constituted the first stage of a broader research project structured into three sequential phases. The present phase focused on device validation, whereas the second phase was planned to include longitudinal data collection and a specific exercise-based intervention over a 6-month period. The third phase was conceived as a pre-post evaluation of the effects of that intervention.

The study was carried out in nursing homes for older adults located in the Plana Baja and Plana Alta areas of the province of Castellón, Valencian Community, Spain. The reference population aged 65 years or older in the province of Castellón is approximately 379,426 inhabitants. The source population for this study was drawn from 809 available places distributed across nine nursing homes. Initial data collection was performed between February and April 2024.

### 2.2. Recruitment, Participants, and Eligibility Criteria

The final sample comprised 151 residents aged 65 years or older. Recruitment began through telephone contact with the participating nursing homes to assess institutional willingness to collaborate and the operational feasibility of the study. Once the centers agreed to participate, face-to-face visits were conducted to present the project, explain the evaluation procedures and review the eligibility criteria.

The inclusion criteria were residence at the facility and age ≥ 65 years, as well as the absence of severe cognitive impairment or total dependence that would prevent the completion of the assessments. Cases were excluded if informed consent was not obtained from the participant or their legal representative, or if the participant was unwilling to participate in the study.

### 2.3. Sample Size Estimation

Sample size was estimated using EPIDAT software, version 4.2. The calculation assumed a sensitivity of 70%, a specificity of 80%, an accuracy of 11%, and an expected prevalence of fall risk of approximately 45–50%. Under these assumptions, the minimum required sample size was 149 participants. The final inclusion of 151 subjects therefore satisfied the estimated sample requirement.

### 2.4. Study Variables and Assessment Tools

The study protocol included the collection of sociodemographic and clinical variables considered relevant for characterizing the sample and for analyzing fall risk and frailty. Sociodemographic data included age, sex, and nursing home of residence. Anthropometric measurements comprised body weight, height, and body mass index (BMI).

### 2.5. Description of the Oldfry Device

The Oldfry device is a portable, low-power instrumental system specifically designed for the objective assessment of movement and functional performance in older adults. The hardware integrates a 9-axis inertial measurement unit (IMU), combining accelerometry, angular velocity (gyroscope), and magnetometry sensors, allowing comprehensive capture of body motion in three-dimensional space.

The system incorporates wireless manual input controls and is powered by a rechargeable battery via micro-USB, facilitating ease of use in clinical and residential settings. The device firmware implements embedded algorithms for real-time acquisition and preprocessing of sensor signals, including configurable filtering and scaling parameters to ensure accurate and stable measurements.

Data acquisition is complemented by a dedicated Android-based application, which enables real-time visualization of movement metrics, execution of motion analysis algorithms, and integration of synchronized video capture. Sensor data are transmitted in real time to a mobile device (smartphone or tablet), allowing combined analysis of movement and recorded video.

The device is positioned at the lower back, at waist level (lumbar region), allowing accurate capture of trunk movements during functional tasks. It operates within a sampling frequency range of 50 to 450 Hz, depending on the configuration settings, ensuring appropriate temporal resolution for movement analysis.

To ensure transparency in the data processing pipeline, the raw inertial signals acquired from the device (triaxial acceleration and angular velocity) were processed using an embedded algorithm to extract clinically meaningful spatiotemporal and kinematic parameters. Signal preprocessing included filtering and segmentation of the different phases of the functional task (sit-to-stand, gait initiation, walking, turning, and sitting down), based on characteristic patterns in the sensor data.

From these segmented signals, key variables were derived, including total task duration, gait speed, turning dynamics, sit-to-stand transition time, and postural stability during static balance conditions. These parameters were subsequently mapped to clinically interpretable outcomes equivalent to the TUG and SPPB scores.

Specifically, the TUG-equivalent value was obtained from the total time required to complete the functional sequence, automatically identified from the inertial signal. For the SPPB, each subcomponent was estimated independently: static balance was quantified using postural sway metrics during the different stance conditions; gait performance was derived from walking speed over the defined distance; and lower limb strength was assessed through temporal and dynamic characteristics of the sit-to-stand movement.

These extracted features were converted into categorical scores according to established clinical thresholds corresponding to TUG and SPPB standard scoring systems. A rule-based algorithm was implemented to classify participants into risk categories, ensuring consistency with conventional clinical interpretation.

**Figure 1:**
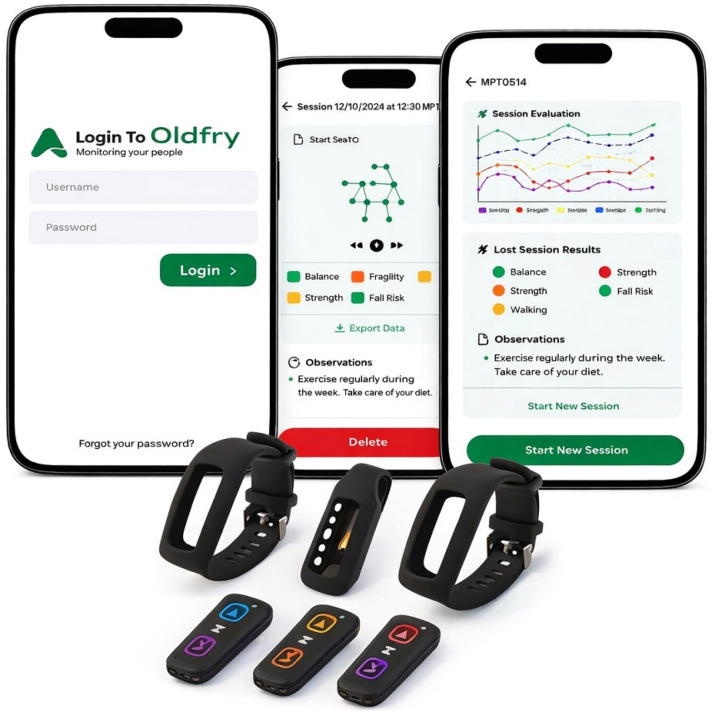
Illustrates the physical structure and sensor configuration of the Oldfry device.

The assessment protocol implemented within Oldfry (Figure 1) incorporates selected components derived from the TUG (0–10: high risk, 10–13.5: mild risk, >13.5: normal risk) [8] and the SPPB (0–3: severe frailty, 4–6: moderate frailty, 7–9: mild frailty, 10–12: normal condition) [9]. Static balance is assessed through three progressive stances (feet together, semi-tandem, and tandem). Subsequently, participants perform a 3 m walk toward a second chair, execute a 180° turn, complete two consecutive sit-to-stand repetitions, and return to the initial chair. All phases of movement are continuously recorded by the inertial sensor.

The processed data are ultimately represented through a standardized color-coded classification system.

Participants underwent a multidimensional assessment based on validated instruments commonly used in geriatric populations. Conventional functional reference measures included the TUG test, used to estimate fall risk, and the SPPB, used to assess physical performance and frailty risk.

### 2.6. Data Collection and Data Management

All assessments were performed by a single investigator to ensure consistency in protocol implementation and to reduce variability associated with multiple evaluators. Data was obtained directly within the nursing homes, under standardized conditions and in the participants’ usual living environment.

To protect confidentiality, each participant was assigned a unique identification code. The correspondence between the code and the participant’s identity was known only to the reference professional at each facility. This procedure ensured anonymity throughout all stages of the study. Data collection included the performance of manual functional tests, together with their corresponding recording through the instrumental device under validation.

### 2.7. Validation Strategy and Statistical Analysis

Validation of the Oldfry device was approached through a diagnostic accuracy framework in which instrumental measurements were compared with the results obtained from the conventional TUG and SPPB assessments.

The ability of the device to discriminate between individuals with and without fall risk was examined using standard diagnostic performance indicators, specifically sensitivity, specificity, positive predictive value, and negative predictive value. To explore the association between the results derived from manual procedures and those generated by the instrumental device, Spearman’s rank correlation coefficient was calculated. In addition, the degree of agreement between both modes of measurement was estimated using the intraclass correlation coefficient (ICC). Taken together, this analytical approach made it possible to assess both the validity and the reliability of the device in the context of fall-risk and frailty assessment in institutionalized older adults.

### 2.8. Ethical Considerations

All participating centers provided their consent to participate in the study after being informed of its objectives and procedures. In addition, the participants or their legal representatives signed the appropriate informed consent form prior to enrollment. The study was conducted in accordance with current legislation regarding data protection, patient autonomy, and biomedical research. Ethical approval was granted by the Ethics Committee of the Universitat Jaume I (UJI) (CEISH/44/2024).

## 3. Results

The final sample of the study consisted of 151 participants, with a median age of 84 years IQR [78.0–91.0]. In total there were 92 women (60.9%) and 59 men (39.1%), of whom 74 (49%) reported experiencing falls. Of those initially contacted, eight were unable to participate: two residents declined participation, and six were unable to complete the test due to severe cognitive impairment.

Regarding the anthropometric variables, the sample consisted of older adults, with a median age of 84.0 years [78.0–91.0]. Median body weight was 64.5 kg [56.7–75.9]. With respect to nutritional status based on BMI classification, normal weight was the most prevalent category (37.1%), closely followed by overweight (36.4%), whereas 25.2% of participants were classified as obese and only 1.3% as underweight. Overall, these findings indicate a nutritionally heterogeneous sample, although most participants were classified as normal weight or overweight. Table 1 compares diagnostic tests for fall risk between the manually administered TUG and the TUG assessed using the Oldfry instrumental device. Regarding reliability, if we consider the positive predictive value (PPV), the manual TUG is 60.4% and the instrumental TUG is slightly higher at 61.8%. These are fairly acceptable figures to establish diagnostic probability. The negative predictive values (NPV) are slightly lower; however, the aim is to ensure that the device measures the outcome of interest, in this case, the probability of falling. Regarding validity, Table 1 shows the sensitivity and specificity values for both manual and instrumental TUG and SPPB. Notably, the instrumental device has a high sensitivity for the SPPB result, practically 91.9%.

Regarding the comparison between the manual SPPB and the instrumental device, the PPV is 51.7% versus 49.3%. For the NPV, it is 61.3% versus 53.8%.

In Table 2, the Spearman correlation coefficients between the manual TUG and the device TUG, as well as between the manual SPPB and the Oldfry instrumental SPPB, can be seen. Reliability coefficients are considered to be those between 0.7 and 0.9. Both the correlation analysis and the concordance are high and within these limits in the comparisons of both measurements

**Figure 2:**
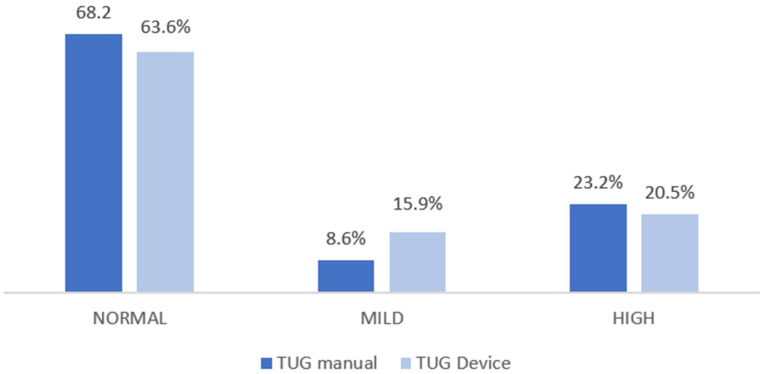
Fall risk assessment (TUG).

Figure 2 establishes a comparison of the percentage of fall risk categorized between the manual and instrumental TUG. It can be seen that the instrumental device reduces the percentage of fall detection among those with normal risk and increases it in the mild risk category compared to the manual TUG.

**Figure 3:**
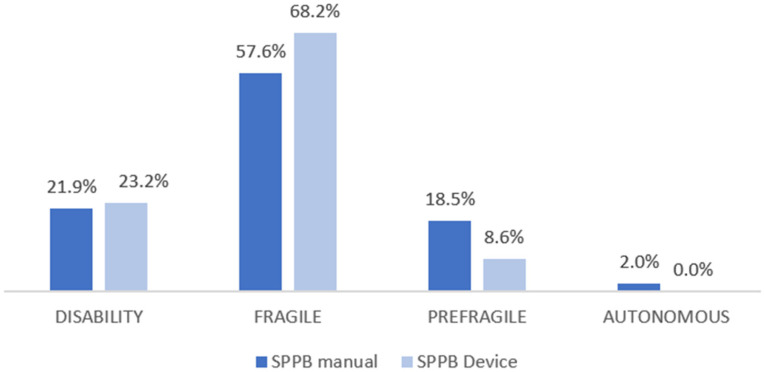
Frailty risk assessment (SPPB).

Figure 3 establishes a comparison of the percentages of frailty risk between the manual and instrumental SPPB. It shows how the instrumental device increases the detection percentage of frailty and disability compared to the manual. It decreases the percentage in the pre-frail and autonomous categories.

## 4. Discussion

This study evaluated the efficacy of the Oldfry instrumental device in comparison with manual measurements performed with common tests like the TUG and the SPPB to determine the risk of falls and frailty in a sample of 151 older adults. Unlike our previous work, which explored the association between clinical and functional variables and fall risk, the present study is specifically designed as a validation study [18].

To validate any instrument, it is crucial that it meets reliability criteria, understood as the property that designates the consistency and precision of the results obtained by an instrument when applied on different occasions. In our study, high linear correlation coefficients were obtained, between 0.7 and 0.9, when comparing manual and instrumental measurements [19].

Additionally, high concordance figures were achieved between the different measurements through the Intraclass Correlation Coefficient, with values above 0.8. Two statistical measures of diagnostic test analysis were included: PPV and NPV, obtaining values above 60% for the TUG and slightly lower for the SPPB.

Validity criteria were also evaluated to ensure that the device effectively measured what it was intended to measure. Specifically, sensitivity criteria were analyzed to identify positive cases and specificity to determine negative cases. The instrumental device demonstrated a specificity of 72.7% for fall risk and a sensitivity of 91.9% for detecting frailty. This differs from the study by Park [20] which found a higher sensitivity for fall risk, at 76% (95% CI: 68–83) and a lower specificity, at 49% (95% CI: 43–54).

The comparison of diagnostic tests between the manual TUG and the Oldfry instrumental device shows that the PPV of the manual TUG is 60.4%, while the PPV of the device is slightly higher, at 61.8%. This finding is consistent with previous studies that have validated other instrumental devices for fall risk assessment. Other authors reported similar PPVs in the validation of electronic tools for fall prediction, highlighting that the accuracy and sensitivity of these devices can match or surpass manual assessments, such as the Johns Hopkins Fall Risk Assessment Tool (JHFRAT) [21].

In other studies [14,21], which included gait speed over 4 m, a PPV225 below 60% was obtained, but a higher NPV, suggesting its usefulness as a screening tool in this context.

Additional research validates frailty tools using questionnaires with various items, such as the study by Bruijnen et al. [22] which validated the G8 tool in oncological patients with surgical indication. This showed lower sensitivity compared to our tool but a higher NPV. Other studies, like Brognara and Vera-Remartínez et al. [12,23], also use inertial sensors to determine fall risk, presenting similar results in terms of sensitivity and slightly higher NPVs. In contrast to previous studies using inertial sensors for fall risk or frailty assessment, the Oldfry device provides a more integrated evaluation by simultaneously estimating both TUG and SPPB outcomes within a single protocol. While earlier approaches have primarily focused on isolated gait parameters or specific functional tasks, our system combines balance, mobility, and lower limb strength into a unified assessment framework.

Concerning the intraclass correlation coefficient of our study, a result above 0.8 was obtained, indicating a high degree of concordance between the measurements, similar to the study by Ye et al., which validated a scale for assessing frailty and showed an adequate intraclass correlation index [24].

Similarly, Mawadda et al. [25] promote a physical activity program to be developed in the community and in the hospital setting to prevent both frailty and fall risk. Thus, the application of a multicomponent exercise program showed improvements in gait speed and gait stability in older adults.

In contrast to previous studies using inertial sensors for fall risk or frailty assessment, the Oldfry device provides a more integrated evaluation by simultaneously estimating both TUG and SPPB outcomes within a single protocol. While earlier approaches have primarily focused on isolated gait parameters or specific functional tasks, our system combines balance, mobility, and lower limb strength into a unified assessment framework. Regarding diagnostic performance, the sensitivity values observed in our study, particularly for frailty detection (91.9%), are comparable to or higher than those reported in similar sensor-based systems, which typically range between 70% and 85% in the previous literature. However, our findings also show lower specificity in some cases, indicating a tendency toward higher detection of positive cases, which may be advantageous in screening contexts but requires consideration in clinical interpretation. Additionally, unlike many smartphone-based or single-task assessment tools, the Oldfry device enables continuous monitoring of multiple functional phases within a single test sequence, providing a more comprehensive representation of patient performance. This integrated approach may enhance ecological validity and facilitate clinical decision-making by offering a multidimensional perspective on fall risk and frailty.

The limitations that should be highlighted in our study are, on one hand, the scarce technological validations to detect both frailty and fall risk since the literature includes numerous studies evaluating a series of items but does not use inertial sensors or biomechanical technology for screening. On the other hand, it is worth noting that the population in which it is developed is mostly dependent due to their advanced age, which in some cases makes participation difficult due to non-compliance with inclusion criteria. Additionally, it is relevant to point out that the main studies with older people involve those over 65 years old, while in our study, most participants are over 80 years old. This age difference could affect the comparability of our results with other studies, as the characteristics and needs of adults over 80 may differ significantly from those of younger older adults in terms of frailty and fall risk within the elderly range.

The main strengths analyzed in our study are the development of a tool capable of combining the detection of both fall risk and frailty in older adults; the ability to identify in which aspect the individual is most lacking to positively and directly impact the physical health of this population sector; and furthermore, the provision to assess fall risk for healthcare professionals through innovation in methodology, structural, usability, and cost terms.

In light of these positive results, the implementation of physical activity sessions in residential nursing homes is anticipated to promote the physical health and autonomy of older adults. Additionally, we plan to develop future research studies to evaluate the long-term benefits of this application, both on an individual and collective level. These future investigations will focus on determining how the continuous use of the Oldfry device can improve the quality of life and functional capacity of users over time.

## 5. Conclusions

The Oldfry device has proven to be an effective tool for detecting both fall risk and frailty in older adults residing in institutions, demonstrating high levels of reliability and sensitivity. This innovative tool not only assesses the overall risk of falls but also allows for the analysis of specific aspects such as balance, mobility, gait, and muscle strength, accurately identifying the areas where each individual has deficiencies.

## Figures and Tables

**Table 1:** Diagnostic tests to assess the risk of falls.

	TUG Manual (CI 95%)		TUG Device (CI 95%)		SPPB Manual (CI 95%)		SPPB Device (CI 95%)	
Sensitivity	39.2	(28.9–50.6)	45.9	(35.1–57.2)	83.8	(73.8–90.5)	91.9	(83.4–96.2)
Specificity	75.3	(64.6–83.6)	72.7	(61.9–81.4)	24.7	(16.4–35.4)	9.1	(4.5–17.6)
Positive Predictive Value	60.4	(46.3–73.0)	61.8	(48.6–73.5)	51.7	(42.8–60.4)	49.3	(41.1–57.5)
Negative Predictive Value	56.3	(46.7–65.5)	58.3	(48.3–67.7)	61.3	(43.8–73.6)	53.8	(29.1–76.8)
% False positives	24.7	(16.4–35.4)	27.3	(18.6–38.1)	75.3	(64.6–83.6)	90.9	(82.4–95.5)
% False Negatives	60.8	(49.4–71.1)	54.1	(42.8–64.9)	16.2	(9.5–26.2)	8.1	(3.8–16.6)
Test accuracy	57.6	(49.6–65.2)	59.6	(51.6–67.1)	53.6	(45.7–61.4)	49.7	(41.8–57.6)
Likelihood ratio (+)	1.59	(0.98–2.57)	1.68	(1.08–2.62)	1.11	(0.95–1.31)	1.01	(0.92–1.11)
Likelihood ratio (−)	0.81	(0.63–1.03)	0.74	(0.57–0.97)	0.66	(0.36–1.20)	0.89	(0.35–2.26)
Pre-test probability (Prevalence)	49.0		49.0		49.0		49.0	

Abbreviations: SPPB; Short Physical Performance Battery. TUG; Time Up and Go.

**Table 2:** Correlation analysis and intraclass correlation coefficient.

	Correlation (Rho S)		Intraclass Correlation Coefficient (ICC)		
	Rho S	Signification	ICC	CI 95%	Signification
TUG manual vs. Device	0.773	0.01	0.821	0.761–0.867	0.0001
SPPB manual vs. Device	0.854	0.01	0.805	0.741–0.855	0.0001

Abbreviations: TUG; Time Up and Go. SPPB; Short Physical Performance Battery. Rho S; Spearman Correlation coefficient.

## Data Availability

The data supporting the findings of this study are available upon reasonable request from the corresponding author. Due to privacy and ethical restrictions, the data are not publicly accessible.

## Abbreviations

The following abbreviations are used in this manuscript:

TUG	Timed Up & Go
SPPB	Short Physical Performance Battery
BMI	Body Mass Index
MNA	Mini Nutritional Assessment
